# *Salvia miltiorrhiza* Bge. (Danshen) for Inflammatory Bowel Disease: Clinical Evidence and Network Pharmacology-Based Strategy for Developing Supplementary Medical Application

**DOI:** 10.3389/fphar.2021.741871

**Published:** 2022-01-19

**Authors:** Siyuan Zhang, Hua Luo, Shiyi Sun, Yating Zhang, Jiaqi Ma, Yuting Lin, Lin Yang, Dechao Tan, Chaomei Fu, Zhangfeng Zhong, Yitao Wang

**Affiliations:** ^1^ Macau Centre for Research and Development in Chinese Medicine, Institute of Chinese Medical Sciences, University of Macau, Taipa, Macao, China; ^2^ School of Pharmacy, Chengdu University of Traditional Chinese Medicine, Chengdu, China

**Keywords:** inflammatory bowel disease, *Salvia miltiorrhiza* bge, danshen, clinical trial, network pharmacology

## Abstract

Inflammatory bowel disease (IBD) is a non-specific colorectal disease caused by multifaceted triggers. Although conventional treatments are effective in the management of IBD, high cost and frequent side effects limit their applications and have turned sufferers toward alternative and complementary approaches. *Salvia miltiorrhiza* Bge (Danshen) is an herbal medicine that reportedly alleviates the symptoms of IBD. A large body of research, including clinical trials in which Danshen-based products or botanical compounds were used, has unmasked its multiple mechanisms of action, but no review has focused on its efficacy as a treatment for IBD. Here, we discussed triggers of IBD, collected relevant clinical trials and analyzed experimental reports, in which bioactive compounds of Danshen attenuated rodent colitis in the management of intestinal integrity, gut microflora, cell death, immune conditions, cytokines, and free radicals. A network pharmacology approach was applied to describe sophisticated mechanisms in a holistic view. The safety of Danshen was also discussed. This review of evidence will help to better understand the potential benefits of Danshen for IBD treatment and provide insights for the development of innovative applications of Danshen.

## Introduction

Inflammatory bowel disease (IBD), including ulcerative colitis (UC) and Crohn’s disease (CD), is a non-specific intestinal disease characterized by chronic inflammation (Olsen et al., 2009). The prevalence rates of UC and CD have risen to 294 and 213 per 100,000 respectively in Europe while the incidence rate of IBD in Hong Kong has tripled to 3.1 per 100,000, illustrating the scale of the disease as a public health issue (Burisch et al., 2013; Mak et al., 2020). Patients with IBD suffer from abdominal pain, diarrhea, hematochezia and weight loss related to pathological changes including ulcerative activation, mucosal damage, and autoimmune disturbance (Petryszyn and Paradowski, 2018). Multi-faceted extrinsic and intrinsic triggers (the latter including microorganisms, genetic susceptibility, and immunological overreaction) are thought to contribute to the initiation and aggravation of IBD. Dyshomeostasis in gut microflora may lead to bacterial invasion through the intestinal barrier and an abnormal immune response with overexpression of inflammatory factors, causing IBD-like symptoms (Lee, 2015). However, the precise etiology is not yet fully understood. Anti-inflammatory agents, such as sulfasalazine and mesalazine, with a long history of utilization, are conventional treatments while biological immunosuppressants, steroids, and even microbiome-inspired therapeutics are effective (Plichta et al., 2019). Although therapeutics of this kind can bring rapid alleviation of symptoms, a limited recovery, high recurrence rates, heavy economic burden, and severe adverse events are major drawbacks and have encouraged sufferers to seek alternative methods for better management of IBD.

Complementary and alternative approaches provide significant benefits for IBD treatment. As a critical part of global supplementary medicine, herbal medicine has attracted much attention due to the unique therapeutic effects of natural material. An abundant body of medical herbs, such as *Coptis chinensis* Franch (Huanglian), *Zingiber officinale* Rosc (Ganjiang), and *Paeonia lactiflora* Pall (Shaoyao), have shown the potentials in the management of colitis (Cao et al., 2019). Among these anti-IBD phytotherapies, Danshen exhibited multilevel efficacies and Danshen-containing products have been utilized in clinic as satisfying treatments. Danshen (*Salvia miltiorrhiza* Bge.) was first recorded in Shennong’s herbal classic of materia medica, Shennong Bencao Jing, a botanical monograph written in the Eastern Han dynasty (AD25-AD220) in China. Danshen is widely applied in the treatment of systemic, especially cardiovascular diseases (Wang L. et al., 2017). More recently, research has shown that natural compounds isolated from Danshen have anti-inflammatory, anti-oxidative and anti-bacterial properties, and are effective in immune regulation and intestinal epithelium protection (Ma et al., 2016; Gao et al., 2017). Danshen extract or molecule compounds could improve colitis symptoms, such as gastro-intestinal bleed, weight loss, and lethal rate in rodent colitis models. In addition, Danshen-based products have shown therapeutic efficacy in clinical trials with attenuation of symptoms and protection from disease recurrence (Deng et al., 2016; Zhu, 2018; Zhu, 2019). Danshen product has been approved by College ter Beoordeling van Geneesmiddelen-Medicines Evaluation Board (CBG-MEB) and used in Europe since 2016.

While these findings imply that Danshen can have a significant impact on IBD, the related molecular mechanisms have not been clearly identified due to methodological limitations related to the “multi-compounds-multi-targets” approach of modeling a sophisticated regulatory network of signal transduction (Luo et al., 2020). Interdisciplinary research has led to the development of identification tools, establishment of a holistic index-assessment system, crosstalk with decussate subjects, and improved statistical analysis methods (Liu et al., 2017; Bai et al., 2018).

This review is a systematic evaluation of the efficacy and safety of Danshen in the treatment of IBD evidenced by experimental studies and clinical trials. Bioactive compounds existing in Danshen were identified, relevant clinical trials were sourced and experimental reports were analyzed to unmask potential mechanisms of Danshen in IBD. To minimize the limitations of the “one-compound-single-target” approach, which does not include the multiple targets in disease, a network pharmacology approach was utilized to estimate and visualize potential actions of Danshen. The safety of Danshen and Danshen-containing products was also discussed. The review provides new ideas about novel applications of Danshen in IBD.

## The Pathology of IBD

IBD is a chronic inflammatory disease induced by multi-faceted triggers including epigenetic factors and genetic susceptibility (Olsen et al., 2009). Smoking is a significant environmental risk factor for exacerbating IBD at any age, including infancy (Shouval and Rufo, 2017), but does not have the same impact in UC patients (Blackwell et al., 2019). Mental stress, health status, infectious diseases, vitamin D deficiency, unhealthy diets characterized by high fat (especially saturated acid), high sugar, high cholesterol, and low fiber, all contribute to the pathogenesis of IBD (Mouli and Ananthakrishnan, 2014; Lewis and Abreu, 2017; Khalili et al., 2018). Antigens and dietary nutrients have an important role in maintaining homeostasis between intestinal bacteria and commensal microflora. For instance, breast-feeding helps to decrease the risks of IBD by improving the homeostatic ecosystem in the infant gut (Andrew R. Barclay et al., 2009). Differences in intestinal microflora structure between patients with IBD and healthy volunteers demonstrated that gut microorganisms are an important factor in the disease, consistent with evidence that animal colitis could be induced in healthy mice by inoculating intestinal microflora from those with UC (Plichta et al., 2019). In addition to the type of gut bacteria, their distribution, abundance, and mobility are important factors in the initiation of IBD (Guo et al., 2020). Fungi, viruses, and parasites also influence gut homeostasis. Interestingly, certain parasites have a beneficial effect on the host immune response, and therefore on colitis (Harris, 2016; Pickard et al., 2017). However, this effect needs to be explored further to provide more compelling evidence on the roles of primary and secondary metabolites in the patho-physiological course of IBD (Segal et al., 2019).

It is well known that invasive pathological bacteria and toxins lead to dysfunction of intestinal epithelial cells and damage in intestinal integrity, which consequently disturbs the host immune response and aggravates inflammatory cascades in mucosal tissues (Katsanos and Papadakis, 2017). The intestinal barrier is a defensive structure composed of host cells, mucosal matrix, synergic bacteria, and immune triggers such as antibodies, antimicrobial peptides, cytokines, and chemokines (Lee, 2015). With the ability to regenerate rapidly in damaged tissues, intestinal epithelial and mesenchymal stem cells have important roles in maintaining mucosal structure and protective functions (Chami et al., 2018). Tight junctions (TJs) between epithelial cells are fundamental to intestinal barrier integrity, and their condition is indicative of the degree of inflammatory damage in IBD. Consequently, mucosal healing is a significant feature of remission in IBD.

IBD involves dysfunction of the innate and adaptive immune systems, with overexpression of inflammatory mediators followed by excessive immune responses inducing chronic or acute inflammatory damage to the colon (Soufli et al., 2016). Macrophages and monocytes play a part in this damage and a range of dysregulated immune cells have been found to disrupt cellular homeostasis (Park et al., 2017). Another notable feature is the abnormal presence of adaptive immune cells including Th1, Th2, Th17, and Treg lymphocytes, which are important to the hosts’ recognition and neutralization of gut antigens (Friedrich et al., 2019). Aberrant expression of pro-inflammatory cytokines such as tumor necrosis factor-α (TNF-α), IL-6, IL-1β, and Interferon-γ (IFN-γ), reflects a persistent disturbed immune response which initiates and aggravates intestinal inflammation (Katsanos and Papadakis, 2017). In patients with UC, the lamina propria and mucosa are infiltrated by Th1 and Th17-profile cells, and a similar process occurs in patients with CD *via* IL-23/IL-17 axis (Feagan et al., 2016; Friedrich et al., 2019). Therapeutic management of intractable IBD requires monoclonal antibodies with high intensity and specific targeting of representative cytokines, and research in this area has increased understanding of chronic inflammatory diseases and their etiologies.

Risk factors for IBD include economic status, lifestyle, diet, society stress, and genetic factors, the latter consistent with evidence of higher risk in certain ethnic or genetic groups (Ng et al., 2013; Eka et al., 2014). Genome-wide gene expression studies have unmasked the existence of a set of IBD-related genes, which participate in different locations and stages of IBD, in parallel with distinctions between UC and CD phenotypes (Noble et al., 2008; Olsen et al., 2009). Mutation of nucleotide binding oligomerization domain-containing protein 2 (NOD2), associated with toll-like receptors (TLRs) and NOD-like receptors (NLRs), is followed by dysfunction of autophagy and inflammatory responses while deletion of mucin 1 (MUC1) and MUC4 disturbs the mucosal homeostasis (Ramanan et al., 2014; Vancamelbeke et al., 2017). Genetic abnormality associated with the host’s immune response, intestinal barrier protection, or inflammatory signal transduction may contribute to the manifestation of IBD-associated symptoms (Eka et al., 2014). However, knowledge of candidate sites is not yet sufficient to evaluate the pathology and to form the basis of diagnostic tests for IBD. Further research is needed to investigate the mechanisms involved. Since holistic outcomes result from sophisticated triggers and interactions at a molecular level, a detailed understanding of molecular mechanisms is important to shed light on prognosis, protection, and amelioration of IBD.

## Phytochemical Characters of Danshen

Phytochemical profiling of Danshen began in the 1930s (Zhou et al., 2005) and enhances understanding of Danshen’s role in disease treatment. With novel extraction, separation, and identification techniques applied in plant research, ingredients have been retrieved from Danshen and their structure are described (Guo et al., 2014). The principal bioactive compounds of Danshen are divided into two parts according to structural characteristics (Zhang XZ. et al., 2016). One is the hydrophilic phenolic acids. The core phenolic structure, in a C_6_C_3_ cluster, exists in both polyphenolic salvianolic compounds like Salvianolic acid B (SAB) and non-polyphenolic compounds such as Salvianic acid A (SA), Caffeic acid (CA), and Protocatechuic aldehyde (PA). The second group, most termed tanshinones, consist of lipophilic phenanthraquinones and their derivatives, is classified into diterpenoids, tricyclic diterpenoids and royleanone tanshinones (Wang L. et al., 2017). Diterpenoid tanshinone, which is represented by Tanshinone IIA (TIIA), Tanshinone IIB (TIIB), and Cryptotanshinone, is constructed according to the basic phenanthraquinone cluster with 1,2-o-quinones core and furan-like units (Hatfield et al., 2013). In addition, the furan or dihydrofuran ring is displaced by isopropyl cluster in tricyclic diterpenoids while royleanone tanshinones are characterized by 1,4-p-quinones, with Miltirone and Isotanshinone respectively.

**FIGURE 1:**
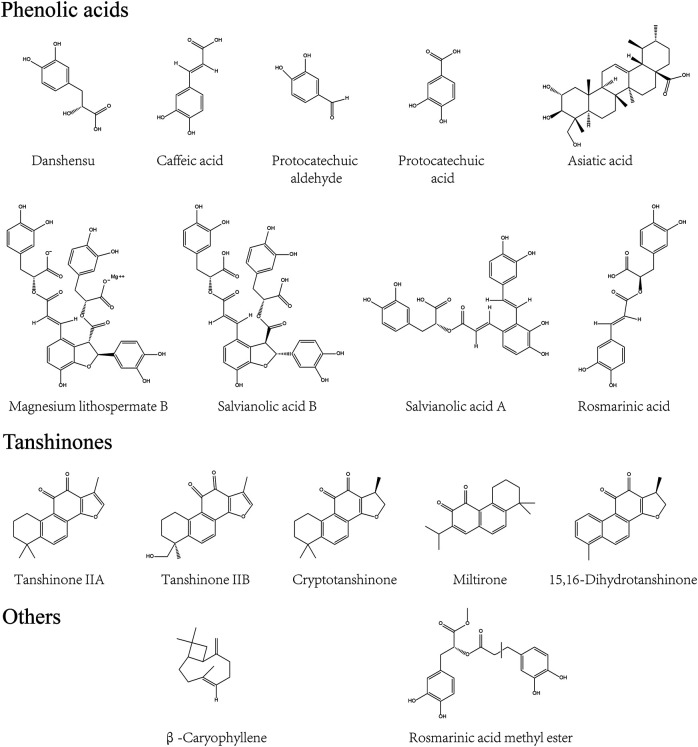
Characteristic compounds of Danshen. Compounds derived from Danshen were divided into phenolic acids, tanshinones and others by their structural characters and pharmacological effects.

Despite differences in structure, compounds of Danshen display common therapeutic effects on IBD, including anti-inflammatory properties of tanshinones and anti-oxidative properties of salvianolic acids (Wang L. et al., 2017; Gao et al., 2017). Other compounds were reported such as triterpenoids and flavones, and including physiological constituents (amino acids and metallic elements) (Guo et al., 2014). In addition, many species of essential oils were discovered in the flower of Danshen rather than the root and rhizome, indicating that novel applications may be found by exploiting all parts of the plant (Liang et al., 2009). However, few reports about non-tanshinone or non-salvianolic acid compounds of Danshen have focused on the management of colitis. For more details on the structure of compounds of Danshen, supplementary reviews are available (Zhou et al., 2005; Zhang XZ. et al., 2016; Wang L. et al., 2017). The compounds outlined above are included in the present review and are depicted in Figure 1.

## Pharmacological Actions and Mechanisms of Danshen for IBD Treatment

### Attenuation of Animal Symptoms in IBD Models With Utilization of Danshen

Since IBD is associated with abnormal immune response caused by multifactorial triggers, it is important to establish experimental models to mimic and investigate this abnormality. Dextran sulfate sodium (DSS) and 2,4,6-trinitrobenzene sulfonic acid (TNBS) disturb the intestinal epithelial barrier and render colonic proteins immunogenic, resulting in a comprehensively dysfunctional immune response including excessive Th1/Th17 activation, cytokine release, and infiltration by immune cells (Kiesler et al., 2015). Compounds of Danshen may protect against DSS and TNBS and thus attenuate animal colitis-indications including weight loss, diarrhea, undereating, shortening colon length, colon weight change, rectal bleeding, diminished health status, alternation in spleen scale, or liver weight change. In addition to these benefits, Danshen was associated with histological changes related to epithelial integrity, the infiltration of neutrophils (marked by CD177) and macrophages (marked by F4/80), T cells and B cell function, and reduction in crypts (Jeong et al., 2015; Jin et al., 2017; Wang et al., 2018).

### Intestinal Epithelial Barrier and Gut Microflora Regulated by Danshen

A feature of IBD is irreversibly degraded integrity of the intestinal epithelial barrier, including TJs classified into Zonula occludens (ZO), Occludins, and claudins (Lee, 2015). Myosin light chain kinase (MLCK), which monitors permeability of the epithelial barrier, interacts with TJs by contraction of the perijunctional actomyosin ring (Feng et al., 2020). Therefore, research has focused on the roles of MLCK in colonic epithelium dysfunction. SAB, the major water-soluble compound of Danshen with anti-inflammatory and anti-oxidative properties, might attenuate abnormality of MLCK after TNBS exposure in rats. Similar to the results *in vivo*, SAB induced microRNA-1 to suppress MLCK detected by dual luciferase reporter assay in IL-1β-stimulated Caco2 intestinal monolayer, consequently ameliorating loss of epithelial ZO-1 and claudin-2 (Xiong et al., 2016; Feng et al., 2020). Similarly, Salvianolic acid A (SAA) attenuated disruption of ZO-1 and Occludin mRNA triggered by DSS (Wang et al., 2018).

Homeostasis of gut microflora is another important factor associated with IBD. While it remains unclear whether bioactive ingredients reverse microbial dysbiosis in the treatment of IBD or ameliorate the inflammatory status to balance microflora distribution, these interactions and their implications for treatment of disease have attracted considerable research interest. For example, SAB and SAA might restore acid balance and promote healthy gut microbiota, with a significant decrease in *Bacteroides* and an increase in *S24-7* (Wu et al., 2018). Consistent with this, enriched microflora populations, particularly *Akkermansia spp*, and improved microbiota balance had been round in DSS-exposed rats treated with SAA and CA (Zhang Z. et al., 2016; Wang et al., 2018). It was of interest that the gut-kidney axis may contribute to regulation of gut microbiota by Magnesium lithospermate B (MLB), suggesting holistic interactions among diverse organs (Zhao et al., 2019b).

### Cell Death Controlled by Danshen

Apoptosis, programmed cell death, acts as a regulatory system in response to extrinsic signals. Morphological and physiological changes during apoptosis can be observed while DNA fragments can be detected by TUNEL assay. The measurement of caspase protein expression, activity, and cleavage-associated maturity, in which B-cell lymphoma 2 (Bcl-2) family members take part, reflects the process of apoptosis (Nagata, 2018). Of note, Caspase-3 is an important mediator in this process. B cells interact closely with cleaved Caspase-3 and Ki-67 while the peroxisome proliferators-activated receptors γ (PPAR-γ) signaling pathway was influenced by the molecular binding of β-Caryophyllene (BC) to Cannabinoid receptor 2 (CB2). In DSS-induced and lipopolysaccharide (LPS)-stimulated experimental models, treatment with BC suppressed expression of macrophage inflammatory protein 2 (MIP2), C-X-C motif chemokine ligand 1 (CINC-1/CXCL1), TNF-α and IL-4 by inhibiting the Nuclear factor kappa B (NF-κB)- extracellular regulated protein kinases (ERK1/2) axis, and attenuated damage due to inflammation (Bento et al., 2011). As cell proliferation and survival are both disturbed in IBD pathogenesis, Rosmarinic acid (RA) was shown to regulate apoptosis-associated mediators including Bcl-XL, Bcl-2, and X-linked inhibitor of apoptosis (XIAP) with its mutually anti-inflammatory performance to obtain remission of DSS-induced colitis. In addition, levels of Cyclin D1 and cyclin dependent kinase 4 (Cdk4), two direct biomarkers for the cell cycle, were also restored to normal after treatment of RA, which protected cellular status when exposed to deleterious factors (Jin et al., 2017). Meanwhile, Tanshinol could protect normal colorectal mucosal cell from apoptosis *via* improvement of low density lipoprotein receptor (VLDR) (Zhu et al., 2021).

Necroptosis is another form of cell death triggered by inflammation and found in the intestinal epithelium of UC patients. When the apoptosis trigger Caspase-8 is blocked, necroptosis regulated by kinases receptor-interacting protein 1 (RIP1)/RIP3/mixed lineage kinase domain-like protein (MLKL) axis is initiated (Tkachenko, 2019). Necroptosis and pyroptosis (an inflammatory programmed cell death) both protect the host from invasive bacteria and feature cell swelling, membrane rupture and cellular content expulsion. NLR family pyrin domain containing 3 (NLRP3) is the most well-studied NLR member to oligomerize apoptosis-associated speck-like protein (ASC), activate Caspase-1 and bring pro-IL-18 and pro-IL-1β to maturation, resulting in Gasdermin-mediated pyroptosis (Kovacs and Miao, 2017). Dihydrotanshinone I (DT) might attenuate DSS-induced damage in the colon and overexpression of pro-inflammatory cytokines through the RIPs-MLKL-Caspase-8 axis, independently of necroptosis (Guo et al., 2018), a property also found in Cryptotanshinone (Min et al., 2020). Asiatic acid (AA) had been found to attenuate colitis symptoms more effectively than sulfasalazine, the therapeutic agent conventionally used in IBD for many years. AA achieved this by scavenging ROS and sustaining mitochondrion membrane integrity, as identified by Mitosox staining and JC-1 staining. Following this attenuation, NLRP3, Caspase-1, and IL-1β were suppressed, as demonstrated both *in vivo* and *in vitro* (Guo et al., 2015). In addition, MLB decreased TNF-α both in DSS-induced chronic and acute colitis with significant suppression of NLPR3/ASC/Caspase-1 pathway (Jiang et al., 2016).

Autophagy is a conserved process in mammals whereby cellular components are degraded and recycled. The ratio of microtubule-associated light chain 3–II (LC3-II) to LC3-I is an important biomarker for this process while other relevant proteins including beclin-1 and Sequestosome 1 (SQSTM1)/p62 play significant roles. It is unclear whether autophagy reduces or increases inflammation, and the interactions of autophagy with the immune response, cell stress, and cellular signal transduction with respect to the etiology of IBD have attracted much attention (Kabat et al., 2016). For example, Tanshinone IIA Sodium Sulfonate (TSS) had shown regulatory effects on LC3-II/LC3-I, beclin-1, and autophagosome, which might contribute to its role in the protection of intestinal epithelium integrity and in downregulation of inflammatory factors including IL-1β, TNF-α, and IL-6 in LPS-induced model (Yang et al., 2018).

### Immunology and Cytokines Induced by Danshen

Although the etiology of IBD is not yet fully understood, evidence increasingly points to a critical role of immunity dysfunction in bacterial penetration of the intestine. Cytokines, which are released by immune cells and a small proportion of non-immune cells, participate in mucosal responsiveness and aggravate inflammatory cascades during the pathological process of IBD (Friedrich et al., 2019). Therefore, anti-inflammatory compounds isolated from Danshen have been tested for their impacts on cytokine-profile etiology characterized by TNF-α, IL-6, IL-1β, IFN-γ and other cytokines. Most of the research on the therapeutic effects of Danshen constituents had focused on pro-inflammatory cytokines as an indicator of disease severity (Zhang Z. et al., 2016; Crespo et al., 2017; Wang et al., 2018).

Cyclooxygenase-2 (COX-2), an immediate-early pro-inflammatory mediator, is induced in response to inflammatory stress with crosstalk of cytokines including TNF-α, IL-1α and IFN-α/β (Jin et al., 2017). After treatment with various Danshen-sourced compounds, the high level of COX-2 was reduced, indicating anti-inflammatory properties of Danshen (Crespo et al., 2017; Jin et al., 2017; Zhao et al., 2018). Similarly, Danshen had protective effects on the multifaceted agents including transcriptional factors and pro- and anti-inflammatory cytokines which trigger the differentiation of T helper cells. Th1 is induced by T-bet and triggers a strong immune response damaging cellular homeostasis, which is suppressed by Th2 and similar cytokines including IL-4 and IL-10. A Danshen patent drug consisting of SA (8%) and PA (16%) might restore dysregulation of T-bet in TNBS-induced murine colitis (Xu Dekui et al., 2012). Certain cytokines have unique properties, such as IL-12 which is essential to activate the development of Th1 cells and release of IFN-γ (Soufli et al., 2016). IL-12 was reduced in DSS-induced mice serum and restored to normal by treatment with CA (Zhang Z. et al., 2016). IL-22 has positive and negative effects, participating both in intestinal mucosal repair and in the inflammation process (Friedrich et al., 2019). Incidentally, RA was manifested to decreased levels of IL-22 in DSS-induced model (Jin et al., 2017). The transcriptional factor forkhead box P3 (Foxp3) is the most reliable biomarker of regulatory T cells (Tregs), responsible for regulating the autoimmune system. The injection of Danshen powder had been found to increase Foxp3 and mRNA in TNBS-induced mouse spleen, enhancing its anti-inflammatory function, specifically its attenuation of MPO (Xu et al., 2014). Of interest, transforming growth factor β (TGF-β) had been found to participate in the management of Foxp3+ Treg cell dynamics, supported by SAA’s regulatory role in inflamed colonic mucosa (Wang et al., 2018). As highlighted by research on the immune regulatory ability of Miltirone, a receptor agonist derived from Danshen (Wang H. et al., 2017), IL-8, an important biomarker of IBD, is overexpressed in damaged intestinal tissues and consequently recruits neutrophils (Liu et al., 2016). Similarly, IL-17, along with chemokines, growth factors, and adhesion molecules form an interactive network which enhances the infiltration of immune cells. The DSS-induced release of pro-inflammatory mediator monocyte chemoattractant protein-1 (MCP-1) or overexpression of IL-17 mRNA might be ameliorated by TIIA (Zhang et al., 2015), and CA respectively, the latter closely correlated with colonic histopathological scores (Ye et al., 2009).

### Free Radicals and Oxidative Stress Balanced by Danshen

In hypoxic conditions, stable hypoxia-inducible factor (HIF)-1α dimerizes with HIF-1β to translocate into cell nuclei and regulate relevant genes for oxidative sensitive mediators and associated proteins such as VEGF which triggers angiogenesis to increase oxygen supply (Perrotta et al., 2015). Rosmarinic acid methyl ester (RAME), a less hydrophilic derivative of RA, suppressed hypoxia inducible factor-prolyl hydroxylase-2 (HPH) and enhanced the release of stable HIF-1α to produce VEGF. This effect formed the basis of RAME treatment to attenuate experimental colitis characterized by pro-inflammatory indicators including MPO, CINC3, COX-2 and inducible nitric oxide synthase (iNOS) (Jeong et al., 2015).

The Nuclear factor E2-related factor 2 (Nrf2) signaling pathway plays an important protective role against inflammation-related oxidative harm marked by reactive oxygen species (ROS), the imbalance of which is regarded as a significant etiological factor for IBD. In this pathway, external stimuli trigger the production of superoxide anion which is transformed into hydrogen peroxide and catalyzed by the catalase/myeloperoxidase (CAT/MPO) enzymatic complex to induce an increase in neutrophils (Balmus et al., 2016). Research had shown that SAB (Xiong et al., 2016), DT (Guo et al., 2018), AA (Guo et al., 2015), RA (Zhao et al., 2018), CA (Ye et al., 2009), and Protocatechuic acid (PAC) (Crespo et al., 2017), suppressed MPO in inflamed colonic tissues, indicating anti-inflammatory and anti-oxidative abilities of Danshen.

Of interest, iNOS and its product nitric oxide (NO), are abundant in inflamed tissues of patients with IBD and in experimental colitis tissue, having alternative impacts which depend on the quantities of NO, stimuli, duration of disease, and even anatomical sites (Soufli et al., 2016). These effects of NO may derive from the reaction with free radical superoxide anion (O2-) to form peroxynitrite (ONOO-) (Kolios et al., 2004). As might be expected, RA, with attenuating effects on colitis syndromes, correlated negatively with NO and iNOS mRNA. In addition, in DSS-induced mice the anti-inflammatory effect of RA, suppressing TNF-α, IL-6 and IL-1β, was enhanced when in combination with black rice anthocyanin-rich extract (Zhao et al., 2018). Other compounds isolated from Danshen including TIIA (Zhao et al., 2018) and PAC (Farombi et al., 2016) also exhibited beneficial impacts on the expression of NO and iNOS. Levels of oxidized-glutathione (GSH), along with relevant enzymes for glutathione metabolism including glutathione peroxidase (GPx) and glutathione-S-transferase (GST), directly reflect cellular oxidative stress. In the DSS-triggered ulcerative colitis model, PAC significantly restored decreased GPx, GST, and Superoxide dismutase (SOD) with resulting reduction in colorectal inflammation. Similar effects also occurred in hepatic tissues indicating that PAC might achieve this outcome *via* complex mechanisms (Farombi et al., 2016). Another report sustained the semblable observation, in which GSSG/GSH ratio, SOD-1, Nrf2, and CAT were reserved (Crespo et al., 2017). In addition, antioxidant properties of SAB might offer protection from TNBS and LPS induced damage, which was noted by NADPH oxidase 4 (NOX4), iNOS, malondialdehyde (MDA), GSH, SOD, and ROS (Xiong et al., 2016).

### Multiple Signaling of IBD Mediated by Danshen

In addition to the aforementioned indicators of IBD severity, cellular transducers form a sophisticated network of signaling pathways in response to extrinsic stress in disease. Research on multiple signaling mediators has the potential to reveal key factors in progression of this disease.

TLRs on the cell membrane surface could recognize stimuli like bacteria and mismatched mediators. When TLR4, one typical kind of TLRs recognizing lipopolysaccharide, is activated, NF-κB would be the fundamental mediator to the initiation and progression of inflammatory diseases like IBD (Mitchell et al., 2016). NF-κB is released after phosphorylation of IκB by IκB kinase (IKK) and then translocates into nuclei to activate promoters of inflammation. Research has shown that compounds of Danshen prevent this release and therefore have a major role to play in limiting inflammation (Nennig and Schank, 2017). Kim et al. reported that, with treatment of 70% ethanol extraction of Danshen, EGFP fluorescence was inhibited in small intestinal segments from *cis*- NF-κB^EGFP^ transgenic mice stressed by LPS, indicating blockage of NF-κB. The inhibitory mechanism was multifactorial and more research was needed to define which bioactive compounds are key to limiting inflammation (Kim et al., 2005). Consistent with the above findings, compared with a DSS-induced model without any treatment, CA suppressed the NF-κB signaling pathway, with decreased levels of nuclear p-p65 and p65, and a decline in IL-6, TNF-α, and IFN-γ. In addition to suppression, balance of gut microflora including *Firmicute*, *Bacteroidetes*, and *Akkermansia* was restored, facilitating intestinal damage remission (Zhang Z. et al., 2016). PAC, the metabolite of Cyanidin-3 β -D-Glycoside (CG), had also been found to inhibit hyperactive NF-κB in a mouse model. With better anti-inflammatory ability than CG, PAC attenuated colitis-associated parameters, including excessive pro-inflammatory triggers (Jang et al., 2016).

As well as hydrophilic components of Danshen, anti-inflammatory properties have also been demonstrated in the lipophilic components, *via* management of NF-κB signaling. Miltirone, an element of tanshinones, downregulated TLR4/NF-κB/IQGAP2 to attenuate colonic inflammation in TNBS-treated mice (Wang H. et al., 2017). Meanwhile, cryptotanshione could inhibit TLR4/p38 MAPK/NF-κB p65 pathway in the colitis (Min et al., 2020).

After phosphorylation at tyrosine residues or serine residues by JAKs, signal transducers and activators of transcription (STATs) accelerate dimerization and consequently translocate into nuclei and activate relevant genes of cytokines and pro-inflammatory enzymes. This contributes to initiating IBD and other chronic inflammatory diseases (Crespo et al., 2017). Synergistically, interactive modification of crosstalk with STATs assists responses to extrinsic and intrinsic factors. RA’s amelioration of DSS-induced colitis derived from decussate suppression of NF-κB and STAT3 signaling transduction characterized by diminished phosphorylation at tyrosine 705 residue rather than serine 727 residue. Following the management of cellular signals, inflammatory mediators including MPO, IL-22, Cdk4, and Cyclin D1 were simultaneously controlled (Jin et al., 2017). As the upstream trigger in STAT3 signaling pathway, Sphingosine-1-phosphate (S1P) and its catalytic synthase, sphingosine kinase (SphK), remain in excess in chronic IBD. PAC had been shown to attenuate excessive SphK1 and S1P receptor (S1P1R) in TNBS-treated mice with impact on S1P-related genes SGPP2 and S1PL. Along with management of the SphK/S1P axis, PAC ameliorated colitis severity depending on antioxidant profile indicated by GSH, CAT, SOD-1, and Nrf2 and intersections with synergistic pathways including AKT, ERK, STAT3, and NF-κB (Crespo et al., 2017).

**FIGURE 2:**
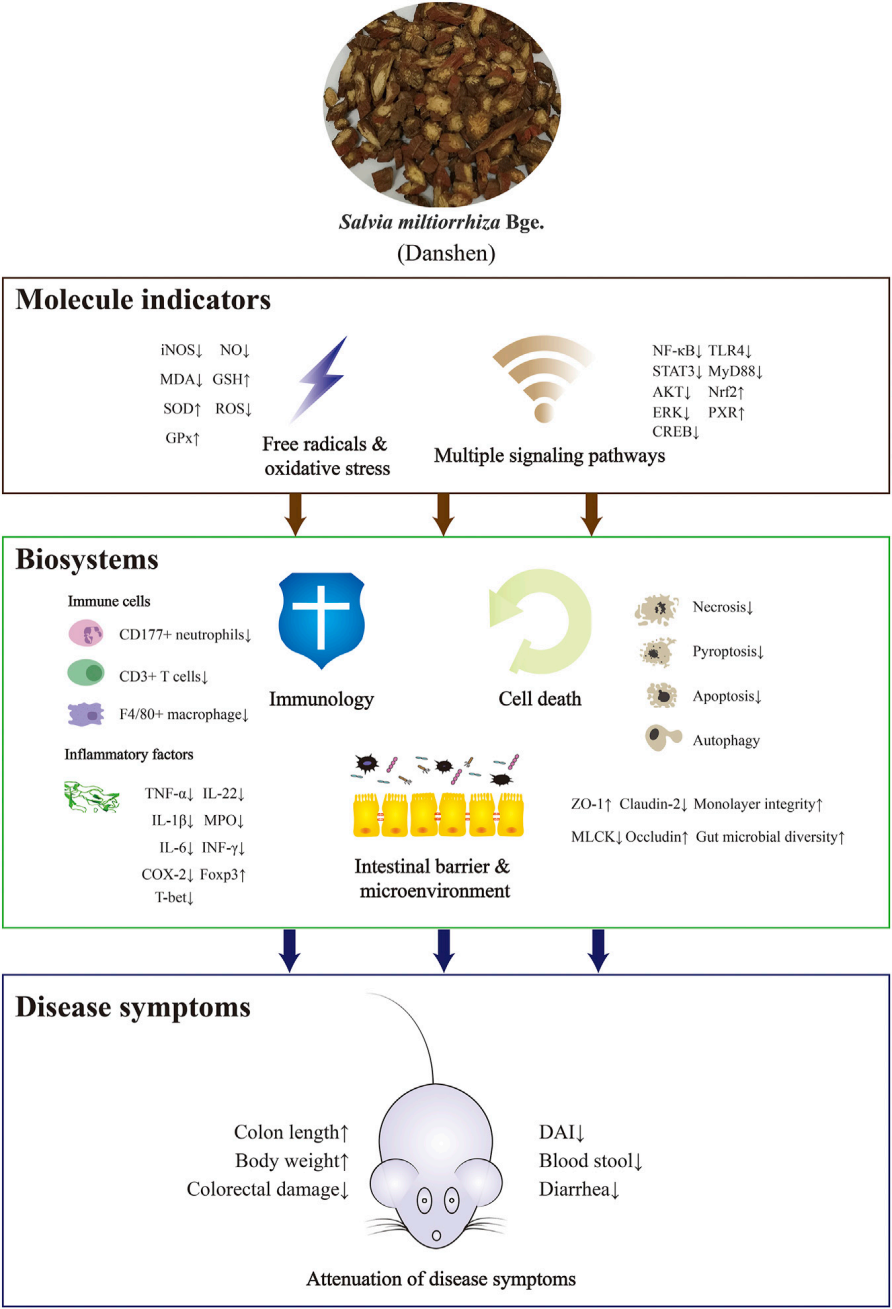
The mechanisms of action of Danshen as a treatment of inflammatory bowel disease (IBD). Danshen exhibited ameliorative effects on IBD among molecular indicators, biosystems and symptoms. The up or down arrows indicated promoting or inhibitory effects of Danshen on the disease indicators.

Intestinal pregnane x receptor (PXR) is inactive in IBD patients, which reflects the necessary role of PXR in maintaining intestinal barrier integrity (Zhang et al., 2015). CYP3A4, CYP4B1, and other xenobiotic-metabolizing genes encoding cytochrome P450 monooxygenases are considered metabolically important in IBD. A compound isolated from Danshen, TIIA, acts as an efficacious PXR agonist. Research on experimental colitis found that levels of metabolic enzymes including CYP3A11, CYP3A13, MDR1, and Gsta1 were restored during disease remission (Zhang et al., 2015). CA restored normal weight in DSS-exposed mice with attenuation of MPO and CYP4B1 expression (Ye et al., 2009; Ye et al., 2011). Other research showing similar regulatory effects suggested CA as a mediator of both pro-inflammatory IL-17 and anti-inflammatory IL-4, indicating multiple functions of CA (Ye et al., 2009). In addition to colonic damage, various organs including liver, kidney, spleen, immune units, and functional systems such as blood coagulation characterized by Plasminogen activator inhibitor-1 (PAI-1) might be adversely affected by pathological changes in IBD. It is therefore of note that PAC ameliorated Aspertate aminotransferase (AST), Alanine transaminase (ALT), and Alkaline phosphatase (ALP), factors indicative of liver toxicity, and restored normal levels of hepatic antioxidant biomarkers including lipid peroxidation, GSH, and H_2_O_2_, resulting in reduced colitis symptoms. However, more evidence is needed to understand the relationship between liver disruption and colitis (Farombi et al., 2016). MLB was found to significantly attenuate symptomatic indices including serum PAI-1, gross bleeding, and damaged mucus and crypt, particularly at a high dose (Jiang et al., 2013). Relevant research is described in Table 1, Table 2, and Figure 2.

**TABLE 1:** Pharmacological details of action of Danshen compounds in experimental colitis.

Compound/Product	Animal model	Colitis symptoms	Immunology and cytokines	Free radicals and oxidative stress	Cell death	Multiple signaling pathways		Intestinal barrier	Microenvironment	Cites
Danshen powder for injection	BALB/C mice (TNBS(100 mg/kg)); BALB/C mice (TNBS(1%) )	DAI↓ Colorectal damage↓	TNF-α↓ T-bet↓ T-bet (mRNA)↓ MPO activity↓ Foxp3 (mRNA)↑ Foxp3↑							Xu Dekui et al. (2012) Xu et al. (2014)
Total phenolic acids + Tanshinones	C57BL/6 mice (DSS (2%))	DAI↓ Colon length↑ Colorectal damage↓	TNF-α↓ IL-6↓ IL-1β↓ COX-2↓			NF-κB↓ TLR4↓ p-PI3K↓ p-AKT↓ mTOR↓				Peng et al. (2020)
Salvianolic acid A	Sprague Dawley rats (DSS (3%))	Body wight↑ Liver weight↓ DAI↓ Colorectal damage↓	TNF-α(mRNA)↓ IL-1β(mRNA)↓ IL-6 (mRNA)↓ TGF-β(mRNA)↓				ZO-1 (mRNA) ↑ Occuldin (mRNA)↑		Gut microbial diversity↑ *Akkermansia* spp*.*↑	Wang et al. (2018)
Salvianolic acid B	C57BL/6 mice (DSS(2.2%)) Sprague-Dawley rats (TNBS/IL-1β); C57BL/6 mice (DSS(4%)) (IL-1β; 200 mg/kg)	DAI↓ Colon length↑ Body weight↑ Colorectal damage↓ Food intake↑	Infiltration: CD3+↓ CD177+↓ F4/80+↓ TNF-α↓ IL-6↓ IL-1β↓ MPO↓	iNOS↓ MDA↓ GSH↑ SOD↑ ROS↓	Apoptosis↓ Bax↓ Bcl-2↑	Production of acetic acid, propionic acid and valeric acid ↑ MLCK↓ miRNA-1↑ NOX4↓	ZO-1↑ Claudin-2↓ Monolayer integrity↑ Occludin↑		*S24-7*↑ *Bacteroides*↓	Xiong et al. (2016), Wu et al. (2018), Feng et al. (2020)
Tanshinol	C57BL/6J mice (5% DSS)	Colon length↑ Colorectal damage↓ Percent survival↑	TNF-α↓ IL-6↓ IL-1β↓			VLDLR↑				Zhu et al. (2021)
Tanshinone IIA sodium sulfonate	C57BL/6J mice (LPS(30 mg/kg))	Colorectal damage↓ Length of jejunum and ileum villus↑	TNF-α↓ IL-6↓ IL-1β↓ TNF-α(mRNA)↓ IL-6 (mRNA)↓ IL-1β(mRNA)↓		LC3-II/LC3-I ↑ Beclin-1↑ Beclin-1 (mRNA)↑ LC3 (mRNA)↑ Autophagosome formation↑					Yang et al. (2018)
Tanshinone IIA	BALB/C mice (DSS( 4%) ); BALB/c mice (TNBS); BC57BL/6 mice (DSS( 3%) )	Diarrhea↓ Bleeding↓ Body weight↑ Colorectal damage↓ Colon length↑ DAI↓	TNF-α(mRNA)↓ IL-6 (mRNA)↓ MCP(mRNA)↓ TNF-α↓ IL-1β↓ MPO↓ Ly6G positive lymphocyte infiltrates↓ MMP-8↓ IL-6↓ IL-10↓	iNOS (mRNA)↓ GSH↑ ROS↓		PXR transactivation↑ DNA-binding by PXR↑ PXR↑ PXR (mRNA)↑ CYP3A4↑ CYP3A4 (mRNA)↑ CYP3A11 (mRNA)↑ CYP3A13 (mRNA)↑ MDR1α(mRNA)↑ GSTα1 (mRNA)↑ NF-κB p65↓	Intestinal permeability↓			Zhang et al. (2015), Bai et al. (2008), Liu et al. (2016)
15,16-Dihydrotanshinone Ӏ	C57BL/6 mice (DSS(5%))	DAI↓Colon length↑ Body weight↑ Colorectal damage↓	TNF-α↓ MPO↓ IL-6↓ COX-2↓ IL-1β↓	iNOS↓	RIP1↓ RIP3↓ caspase-8↑ MLKL↓	HMGB1↓				Guo et al. (2018)
Cryptotanshinone	C57BL/6 mice (DSS(4.5%))	DAI↓ Colon length↑ Body weight↑ Colorectal damage↓	TNF-α↓ MPO↓ IL-6↓ IL-1β↓ COX-2↓	iNOS↓						Min et al. (2020)
Miltirone	C57BL/6 mice (TNBS (100 mg/kg))	DAI↓ Colon length↑ Body weight↑ Colorectal damage↓	TNF-α↓ IL-6↓ IL-1β↓ IL-8↓			TLR4↓ MyD88↓ NF-κB p65↓ TLR4 (mRNA)↓ MyD88 (mRNA)↓ IQGAP2↑ IQGAP2 (mRNA)↑				Wang et al. (2017a)
Rosmarinic acid	ICR mice (DSS(5%)); C57BL/6 mice (DSS(3%))	Body weight↑ DAI↓ Spleen weight↓ Colorectal damage↓	MPO↓ IL-6↓ IL-1β↓ IL-22↓ COX-2↓ TNF-α↓ COX-2 (mRNA)↓ TNF-α(mRNA)↓ IL-6 (mRNA)↓ L-1β(mRNA)↓	iNOS↓ NO↓ iNOS(mRNA)↓	Survivin↓ Bcl-2 ↓ Bcl-xL↓ XIAP↓ Cdk4↓ Cyclin D1↓	NF-κB p65↓ p65 translocation↓ p-IκBα↓ IκBα↑ p-STAT3↓ p-STAT3 translocation↓				Jin et al. (2017), Zhao et al. (2018)
Rosmarinic acid methyl ester	Rats (TNBS)	Colorectal damage↓	MPO↓ CINC-3↓ iNOS↓ COX-2↓	HIF-1α↑ Stabilization of HIF-1α↑ Inhibition of HPH↑ VEGF↑						Jeong et al. (2015)
Asiatic acid	C57BL/6 mice (DSS(2.5%))	DAI↓ Colon length↑ Body weight↑ Colorectal damage↓	TNF-α↓ IL-6↓ IL-1β↓ MPO↓ IFN-γ↓ TNF-α(mRNA)↓ IL-6 (mRNA)↓ IL-1β(mRNA)↓ IFN-γ(mRNA)↓		Caspase-1 activation↓ Caspase-1 p10↓					Guo et al. (2015)
β-Caryophyllene	CD1 mice (DSS(3%))	DAI↓ Colon length↑ Body weight↑ Colorectal damage↓	TNF-α(mRNA)↓ IL-1β(mRNA)↓ CXCL1/KC(mRNA)↓ IFN-γ(mRNA)↓ IL-4↑ MPO activity↓ Foxp3 (mRNA)↑ TNF-α↓ IL-1β↓ CXCL1/KC↓ IFN-γ↓ MIP-2↓			CB2(mRNA)↓ CINC-1 (mRNA)↓ PPARγ(mRNA)↑ NF-κB p65↓ p-CREB↓ p-ERK↓ p-IKKα/β↓ Cleaved caspase-3↓				Bento et al. (2011)
Protocatechuic acid	BALB/C mice (TNBS); Wistar rat (DSS(5%)); ICR mice (TNBS(2.5%))	Body weight↑ Colorectal damage↓DAI↓ Colon length↑	MPO↓ TNF-α↓ IL-6↓ IL-1β↓ COX-2↓ TNF-α(mRNA)↓ IL-6 (mRNA)↓ IL-1β(mRNA)↓ COX-2 (mRNA)↓	Nrf2↑ MDA ↓ GSH↑ SOD↑ CAT↑ GST↑ GPx↑ H_2_O_2_↓ iNOS↓ NO↓		SphK1↓ S1PR1↓ SphK1 (mRNA)↓ S1PR1(mRNA)↓ S1PL (mRNA)↑ SGPP2(mRNA)↓ S1P↓ p-STAT3↓ p-AKT↓ p-ERK1/2↓ NF-κB p65↓ AST↓ ALT↓ ALP↓				Crespo et al. (2017); Farombi et al. (2016); Jang et al. (2016)
Magnesium lithospermate B	BALB/C mice (DSS(5%))	Body weight↑ Colorectal damage↓ Microvilli integrity ↑	CD40/CD40L↓ IL-6↓ CD40 (mRNA)↓ CD40L (mRNA)↓		ASC↓ NLRP3↓ Caspase-1↓	PAI-1↓				Jiang et al. (2013), Jiang et al. (2016)
			TNF-α↓							
Caffeic acid	C3H/HeOuJ mice (DSS(1.25%)); C57BL/6 mice (DSS(2.5%))	Food intake↓ Colon length↑ Body weight↑ Colorectal damage↓ DAI↓	MPO↓ IL-17 (mRNA)↓ IL-4 (mRNA)↑ IL-6↓ TNF-α↓ IFN-γ↓ IL-12↑ Infiltration of CD3^+^ T cells↓ CD177 + neutrophils↓ F4/80 + macrophage↓	iNOS(mRNA)↓		Cyp4b1 (mRNA)↑ Cytoplasmic IκBα↑ p65 translocation↓			*Akkermansia*↑	Ye et al. (2009), Zhang et al. (2016b)

PAI-1, Plasminogen activator inhibitor type-1; AST, aspertate aminotransferase; ALT, alanine transaminase; ALP, alkaline phosphatase; Cebpb, CCAAT/enhancer-binding protein and beta; Hp, haptoglobin; S100a8, S100 calcium binding protein a8; Saa3 = serum amyloid A3; DAI, disease activity index.

**TABLE 2:** Pharmacological details of action of Danshen compounds *in vitro* or *ex vivo*

Compound/Product	Cell types	Stimulus	Doses of danshen compound/product	Mediated biomarkers	Cites
Danshen water-soluble extract	IEC-18 Small intestinal segments Primary intestinal epithelial cells	LPS, 10 μg/ml	100, 250, 500 μg/ml	ICAM-1 (mRNA)↓, NF-κB↓ p-IκBa↓ IκBa↑ p-p38↑ IKK activity (directly)↓ NF-κB transcriptional activity↓ RELA nuclear translocation↓ NF-κB recruitment to the ICAM-1↓	Kim et al. (2005)
Salvianolic acid B	Caco-2 intestinal monolayer Raw264.7	IL-1β, 10 ng/ml	20, 40, 80 µM	ROS↓MLCK↓ miRNA-1↑ZO-1↑ Claudin-2↓ Monolayer integrity↑	Xiong et al. (2016)
Tanshinol	Normal colorectal mucosal cell	LPS, 8 μg/ml	25, 50, 100 μM	IL-8↓ IL-1β↓ IL-6↓ IL-8 (mRNA)↓ IL-1β(mRNA)↓ IL-6 (mRNA)↓ cell apoptosis↓ Bax↓ Bcl-2↑ Cleaved-Caspase 9↓ Cleaved-Caspase 3↓ VLDLR↑ VLDLR (mRNA)↑	Zhu et al. (2021)
Tanshinone IIA	LS174T Peripheral blood neutrophils	LPS, 1 μg/ml	2.5, 5, 10, 20 μM; 50 μM	TNF-α↓ IL-1β↓ MPO↓ IL-6↓ IL-10↓ neutrophil migration↓ ROS↓ PXR transactivation↑ DNA-binding by PXR↑ PXR↑ PXR (mRNA)↑ CYP3A4↑ CYP3A4 (mRNA)↑ CYP3A11 (mRNA)↑	(Zhang et al., 2015); Liu et al. (2016)
15,16-Dihydrotanshinone Ӏ	RAW 264.7 HT-29	LPS, 1 μg/ml TNF, 20 ng/ml + BV6, 0.5μM + ZVAD 20 μM	0.1, 0.2, 0.5 μM	TNF-α↓ IL-6↓ COX-2↓ iNOS↓ Nitrite↓p-RIP1↓ p-RIP3↓ caspase-8↑ p-MLKL↓	Guo et al. (2018)
Cryptotanshinone	RAW 264.7 HT-29	LPS, 1 μg/ml TNF, 20 ng/ml + BV6, 0.5μM + ZVAD 20 μM	2, 5, 10 μM	TNF-α↓ IL-6↓ COX-2↓ iNOS↓ RIP3↓ TLR4↓ p-p65↓ p-p38↓ p-ERK1/2↓ p-SAPK/JNK↓	Min et al. (2020)
Miltirone	IEC-6	LPS, 0.5 mg/L	5, 15, 50 μM	TLR4↓ MyD88↓ NF-κB p65↓ TLR4 (mRNA)↓ MyD88 (mRNA)↓ IQGAP2↑ IQGAP2 (mRNA)↑	Wang et al. (2017a)
Asiatic acid	THP-1 Peritoneal macrophages	LPS, 100 ng/ml + ATP, 5 mM	15, 30, 60 μM	IL-1β↓Mitochondrial ROS generation↓Caspase-1 activation↓ Caspase-1 p10↓ NLRP3 inflammasome formation ↓	Guo et al. (2015)
β-Caryophyllene	IEC-6 Lymph Node Macrophage Marrow-Derived Macrophage	LPS, 10 μg	10 μM	TNF-α↓ IL-1β↓ CXCL1/KC↓	Bento et al. (2011)
Protocatechuic acid	Peritoneal macrophage	LPS, 100 ng/ml	10 μM	TNF-α↓ NF-κB p65↓	Jang et al. (2016)

## Clinical Trials of Danshen as a Treatment of UC

Danshen is traditionally used for menstrual disorder, traumatic injury, anxiety, depression, and cardiovascular diseases. Recent investigations have reported that Danshen and its constituents may have potential for protection against cancer, inflammation, allergy, oxidation and harmful microorganisms, and may have a role in immune regulation (Gao et al., 2017; Gu et al., 2017; Li et al., 2018). Evidence-based therapeutic effects on diabetes, autoimmune disease, alcohol dependence, and nervous system disorders such as Parkinson’s disease and Alzheimer’s disease suggest the scope for a range of clinical applications, which sustain the Danshen-based pharmaceutic industry (Yin et al., 2009; Su et al., 2015).

Two or more participant herbs with different therapeutic functions are combined to create a formula with the potential to treat complex clinical disorders. In Chinese herbal medicine, a core characteristic herb within this combination determines the therapeutic direction of the formula. Danshen is introduced in Chinese herbal medicine theory as a medicine to treat blood-associated diseases (Liao et al., 2019), and reports of its effectiveness as a treatment for IBD and related syndromes are rare. However, medicinal formulae in which Danshen is the main constituent are increasingly applied to the management of IBD. In addition, many clinical trials using Chinese patent drugs mostly consisting of Danshen such as Danshen Injection have been reported.

In this review, papers describing clinical trials on the effectiveness of Danshen for the treatment of UC, the most frequently occurring phenotype of IBD, were sought from the following databases: China National Knowledge Infrastructure (CNKI), Chinese Science and Technology Periodical database (VIP), Wanfang, Chinese biomedical literature service system (SinoMed), and other resources in Chinese or in English based on Pubmed, MEDLINE, embase, and the Cochrane Central Register of Controlled Trials. To build a complete figure, “Danshen”, “*Salvia miltiorrhiza* Bge.“, and “ulcerative colitis” were regarded as search terms without limitations. Then, only randomized controlled trials (RCTs) till Dec. 2020 without significant bias assessed by Cochrane criteria and in which patients were diagnosed with UC were included in the review.

**FIGURE 3:**
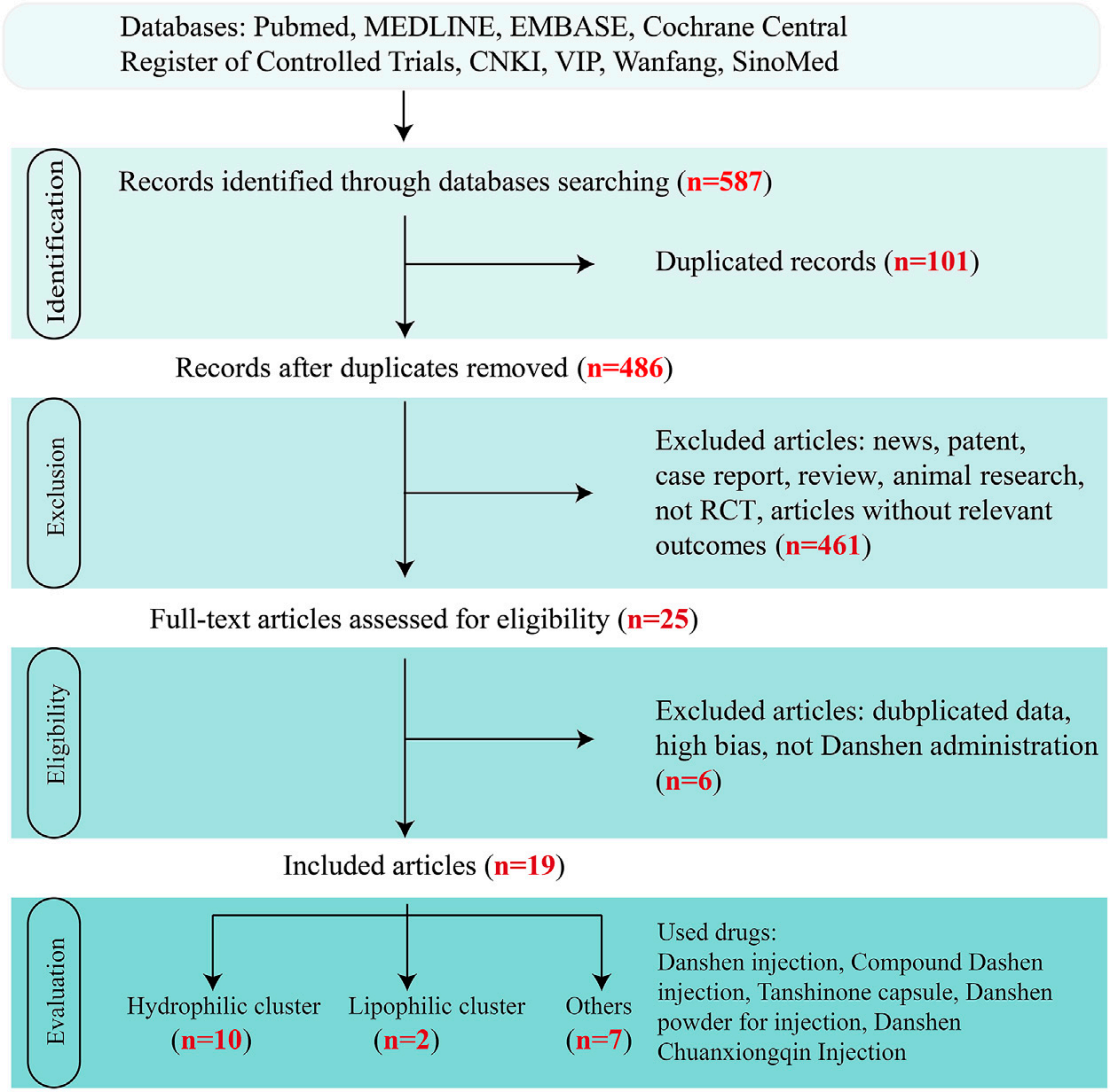
Flow diagram of study selection of clinical trials. Clinial records on Danshen products for inflammatory bowel disease (IBD) were screened through the steps of identification, exclusion, eligibility, and evaluation.

Overall, 19 clinical trials with a total of 1,440 patients were included in Figure 3. Although diagnostic criteria varied between these trials, patients who were eligible generally exhibited typical symptoms of UC characterized by hematochezia, abdominal pain, and consistent diarrhea. Only two trials used the Mayo Score to assess the disease course (Deng et al., 2016; Liu et al., 2019) while 5 trials recorded the precise location of ulcers, such as descending colon, sigmoid colon and rectum (Dong, 2004; Zhu, 2018; Zhu, 2019) and at least eight trials described the pathological course of UC including severity and remission phases (Han et al., 2005; Yang and Zhang, 2005). No significant heterogeneity in age, treatment course, disease course, gender, or ethnicity was found among the trials. Danshen-based interventions consisted of Compound Danshen injection (Li and Luo, 2011; Zheng et al., 2013), Danshen injection (Zheng et al., 2013), Danshen powder for injection (Li, 2019), Tanshinone capsule (Xiong and Wang, 2016; He, 2018), Danshen Chuanxiong injection (Sheng et al., 2014; Xu, 2015). Characteristics of these products including pharmaceutical forms, dose, frequency, and mode of administration were shown in Tables 3, 4, 5. All interventions in experimental groups were combinations of Danshen products and conventional treatments for UC including sulfasalazine, mesalazine, probiotics, antibiotics, or dexamethasone and these were compared with only the conventional treatments in the control groups.

**TABLE 3:** Clinical trials of Danshen products as supplementary interference in the regulation of ulcerative colitis (UC) (Hydrophilic cluster).

Study design	Interventions	Approach, duration, dose, frequence of danshen treatment	Participants	Amount	Information of patients *m*, f, age (year), course (year)	Clinical efficacy (C/T)	Adverse events	Outcome measurements	References
RCT	C:mesalazine T:mesalazine, Danshen powder for injection	i.v.gtt, 8w, 0.4 g, qd	C:51 T:51	102	C: N, N, N, N T: N, N, N, N	78.43%/94.12%	C: 4, abdominal pain, nausea, diarrea T: 7, abdominal pain, nausea, diarrea	TNF-α, IL-6, IL-8, Hcy	Li, (2019)
RCT	C:mesalazine T:mesalazine + Danshen injection	i.v.gtt, 4w, 10ml, qd	C:27 T:27	54	C: 16, 11, 39.84 ± 9.68, 2.75 ± 1.28 T: 15, 12, 38.53 ± 10.37, 2.51 ± 1.74	70.37%/96.30%	C: 4, hash, nausea, diarrea T: 3, hash, diarrea	TNF-α, IL-6, IL-8	Zhu, (2018)
RCT (random number table)	C:probiotics + mesalazine T:probiotics + mesalazine + Danshen injection	i.v.gtt, 6m, 20ml, qd	C:46 T:47	93	C: 25, 21, 33.17 ± 6.50, 4.88 ± 3.12 T: 24, 23, 34.2 ± 6.46, 4.90 ± 3.09	73.91%/93.62%	C: 3, nausea, dizziness T: 5, nausea, localized edema, hypotension	d-lactate, DAO, QOL score	Yang, (2017)
RCT	C:probiotics + mesalazine T:probiotics + mesalazine + Danshen injection	i.v.gtt, 4w, 20 ml,qd	C:32 T:32	64	C: 18, 14, 39.9 ± 6.5, N T: 17, 15, 40.4 ± 8.5, N	N	C: 6 T: 8	defecating frequency, haemorrhoids blood in stool, Mayo Score, Blood sendimentation, PLT	Liu et al. (2019)
RCT	C:ornidazole + sulfasalazine T:ornidazole + sulfasalazine + Danshen injection	i.v.gtt, 4w,20ml, qd	C:30 T:30	60	C: 22, 8, 47.9 ± 8.1, 3.2 ± 0.7 T: 20, 10, 48.1 ± 7.9, 3.1 ± 0.6	53.30%/83.30%	C: 1, headache T: 1, headache	DAI, CRP, Blood sendimentation	Yao et al. (2016)
RCT	C:mesalazine T:mesalazine + Danshen injection	i.v.gtt, 4w,20ml, qd	C:49 T:49	98	C: 29, 20, 37.6 ± 5.8, 4.2 ± 0.8 T: 28, 21, 37.4 ± 5.7, 4.2 ± 0.9	77.55%/93.88%	C: N T: N	TNF-α, IL-8, PLT, FIB	Zhu, (2019)
RCT (random number table)	C:mesalazine T:mesalazine + Danshen injection	i.v.gtt, 4w,20 ml+	C:55 T:55	110	C: 23, 32, 57.6 ± 7.5, 4.5 ± 1.3 T: 25, 30, 58.3 ± 8.2, 4.8 ± 1.4	80.00%/94.50%	C: N T: N	TNF-α, IL-6, IL-8, FIB, PLT, MPV, Recurrence rate	Deng et al. (2016)
RCT (random number table)	C:mesalazine T:mesalazine + Danshen injection	i.v.gtt, 4w,20ml, qd	C:43 T:43	86	C: 25, 18, 45.4 ± 1.3, 2.4 ± 0.3 T: 23, 20, 45.6 ± 1.2, 2.3 ± 0.4	81.40%/95.40%	C: 5, nausea, fever, hash T: 3, nausea, fever, disturbance in respiration	CRP, Blood sendimentation, d-lactate	Sun, (2019)
RCT	C:mesalazine T:mesalazine + Danshen injection	i.v.gtt, 4w, 4ml, qd	C:36 T:36	72	C: 19, 17, 44.6 ± 2.5, 3.2 ± 1.2 T: 20, 16, 44.2 ± 2.7, 3.3 ± 1.4	77.78%/94.44%	C: 4, nausea, diarrhea, hash T: 2, diarrhea, hash	IL-8, IL-10, TNF-α, FIB, PLT, MPV	Du, (2019)
RCT	C: sulfasalazine T:mesalazine + Danshen injection	i.v.gtt, 4w, 4ml, qd	C:50 T:50	100	C: 26, 24, 36.6 ± 12.7, 2.1 T: 27, 23, 36.8 ± 11.3, 2.3	62.00%/84.00%	C: N T: N	D-dimer, FIB, blood viscosity	Huang, (2005)

RCT, random control trial; i.v.gtt, intravenously guttae; qd, quaque die; Hcy, homocysteine; DAO, diamine oxidase; DAI, disease activity index; CRP, C-reactive protein; PLT, serum levels of platelet; FIB, plasma fibrinogen; MPV, mean platelet volume; N, not available; T, danshen treatment; C, conventional treatment.

**TABLE 4:** Clinical trials of Danshen products as supplementary interference in the regulation of ulcerative colitis (UC) (Lipophilic cluster).

Study design	Interventions	Approach, duration, dose, frequence of danshen treatment	Participants (T/C)	Amount	Information of patients n, m/f, age (year), course (year)	Clinical efficacy	Adverse events	Outcome measurements	References
RCT (random number table)	C:mesalazine T:mesalazine + Tanshinone capsule	p.o., 4w, 1g, tid	C:44< T:44	88	C: 26, 18, 45.88 ± 9.22, 7.21 ± 5.30 T: 24, 20, 44.56 ± 10.11, 8.06 ± 4.83	N	N	PLT, MPV, APTT, FIB, D-D, CRP, TF, TFPI	He, (2018)
RCT (random number table)	C:mesalazine T:mesalazine + Tanshinone capsule	p.o., 8w,1g, tid	C:35 T:35	70	C: 15, 20, 46.8 ± 7.1, 5.2 ± 2.1 T: 16, 19, 47.4 ± 6.2, 5.1 ± 2.3	N	N	CRP, TNF-α, IL-6, APTT, FIB, PLT, MPV, PAG, D-D	Xiong and Wang, (2016)

N, no information; RCT, random control trial; CRP, C-reactive protein; PLT, serum levels of platelet; FIB, plasma fibrinogen; MPV, mean platelet volume; p.o, per os; tid, three times daily; PAG, platelet aggregation; APTT, activation partial prothrombin time; FIB, fibrinogen; D-D, D-dimer; CRP, C-reactive protein.; m: male; f:female; T: danshen treatment; C:conventional treatment.

**TABLE 5:** Clinical trials of Danshen products combined with other Chinese herbal products as supplementary interference in the regulation of ulcerative colitis (UC).

Study design	Interventions (T/C)	Approach, duration, dose, frequence of danshen treatment	Mayor constituents of danshen products	Treatment course (day)	Amount	Participant amount (T/C) T: M, F, age, duration of disease; C: M, F, age(y), duration of disease(y)	Clinical efficacy (T/C)	Outcome measurements	Adverse events	References
RCT	Fufang Danshen Injection + Sulfasalazine/Sulfasalazine	en, 40ml, bid	Salvianic acid A, Protocatechuic aldehydrate, Salvianolic acid B	28	63	32/31; T: 14, 18, 43.5 ± 2.7,N/C: 15, 16, 42.3 ± 2.5, N	96.9%/61.3%	N	N	Li and Luo, (2011)
RCT (registration order)	Fufang Danshen Injection + Sulfasalazine + Tinidazole + Dexamethasone/Sulfasalazine + Tinidazole + Dexamethasone	i.v.gtt, 24ml, qd	Salvianic acid A, Protocatechuic aldehydrate, Salvianolic acid B	28	48	23/25; T: N, N, N, N/C: N, N, N, N	86.9%/84.0%	Relapse rate	T: N/C: 32% (8) nausea, burning sensation, liver dysfunction	Dong, (2004)
RCT (random number table)	Fufang Danshen Injection + Sulfasalazine/Sulfasalazine	ivqtt, 20ml, qd	Salvianic acid A, Protocatechuic aldehydrate, Salvianolic acid B	20	60	30/30; T: 17, 13, 34.5 ± 4.8, 20.8 ± 7.4m/C: 15, 15, 35.9 ± 5.3, 27.6 ± 6.9m	90%/76.67%	Endoscopic remission; Relapse rate	N	Zheng et al. (2013)
RCT	Danshen Injection + Huangqi Injection + Dexamethasone/Dexamethasone	i.v.gtt, 40–70ml, qd	Salvianic acid A, Protocatechuic aldehydrate, Salvianolic acid B, Astragaloside Ⅳ, Astragaloside Ⅲ, Astragaloside Ⅰ	28	56	26/30; T: 15, 11, 39,N/C: 16, 14, 40, N	N	Duration of symptoms; Minimum dose of glucocorticoid	N	Yang and Zhang, (2005)
RCT	Danshen Injection + Huangqi Injection + Dexamethasone + Conventional treatments/Dexamethasone + Conventional treatments	i.v.gtt, 0.8–1.2 ml/kg, qd	Salvianic acid A, Protocatechuic aldehydrate, Salvianolic acid B, Astragaloside Ⅳ, Astragaloside Ⅲ, AstragalosideⅠ	28	84	46/38; T: N, N, N/C: N, N, N	N	Duration of symptoms; Minimum dose of glucocorticoid	T: N/C: 13.2% (5) interstinal perforation, worsen condition	Han et al. (2005)
RCT (random number table)	Danshen Chuanxiongqin Injection + Sulfasalazine/Sulfasalazine	i.v.gtt, 5ml, qd	Salvianic acid A, Protocatechuic aldehydrate, Isoferulic acid, Rosmarinic acid, Salvianolic acid A, Ligustrazine hydrochloride	28	86	43/43; T: 27, 16, 45.9 ± 9.2, 3.6 ± 0.7 years/C: 26, 17, 46.3 ± 9.6, 3.2 ± 0.6 y	95.3%/74.4%	Symptom scores	T: 16.3% (7)/C: 18.6% (8) gastrointestinal response, rash, headache gastrointestinal response, rash, headache	Xu, (2015)
RCT (registration order)	Danshen Chuanxiongqin Injection + Clostridium Butyricum Capsule + Methalazine/Clostridium Butyricum Capsule + Methalazine	i.v.gtt, 10ml, qd	Salvianic acid A, Protocatechuic aldehydrate, Isoferulic acid, Rosmarinic acid, Salvianolic acid A, Ligustrazine hydrochloride	28	46	28/18; T: 17, 11, 43.2, 1.8 years/C: 10, 8, 42.6, 2.1 y	89.3%/83.3%	Duration of symptoms; Blood sendimentation; CRP	T: 17.9% (5)/C: 16.7% (3) abdominal distension, nausea, rash abdominal distension, nausea, rash	Sheng et al. (2014)

N, no information; RCT, random control trial; CRP, C-reactive protein; en, enema; i.v.gtt, intravenously guttae; qd, quaque die; bid, bis in die; T, danshen treatment; C, conventional treatment.

The rate of clinical efficacy attributed to Danshen plus conventional treatment of UC ranged from 83.3 to 96.9%, suggesting that either aqueous or lipophilic constituents of Danshen exhibited therapeutic effects. Although 11 trials reported adverse events including rash, headache, diarrhea, nausea, abdominal pain, and dizziness (Han et al., 2005; Sheng et al., 2014; Liu et al., 2019), these side effects were ameliorated after cessation of treatment. Similar rates of adverse events between experimental groups and control groups suggested that Danshen is a safe treatment.

In the patients taking Danshen products, both duration and severity of clinical symptoms including abdominal pain, blood stool, diarrhea, and tenesmus were ameliorated. Endoscopic evaluation of mucosal condition showed improvement with less erosion, better healing in ulcers, and improvement in microenvironment ecosystem, and the recurrence of UC was decreased. The following biomarkers representing inflammation and microcirculation were also at healthy levels: IL-6, IL-8, IL-10, TNF-α (Zhu, 2018; Li, 2019), C-reactive protein (CRP), d-Lactate (DL), D-dimer (D-D), blood sedimentation, serum levels of platelet (PLT), plasma fibrinogen (FIB), and mean platelet volume (MPV) (Xiong and Wang, 2016; He, 2018). Based on the collective clinical evidence, Danshen was considered as a potential auxiliary treatment for UC.

The clinical evidence summarized above suggests that Danshen may be considered as a potential auxiliary treatment for UC. However, while the included studies were randomized and controlled, they were found to be of low quality due to methodological flaws including small samples, and potentially biased selection processes. Hence, more standardized clinical trials of randomized, double-blind, and multi-centered design are needed to further test the safety and efficacy of Danshen products.

## Network Pharmacology Approach Unmasking the Holistic Mechanism of Danshen

In contrast to conventional medicine, the holistic nature of Chinese herbal medicine is characterized by multi-compounds, multi-targets, multi-functions, and multi-systems, acting co-operatively toward a desired outcome. This network model challenges researchers to explain its precise mechanisms of action. Network pharmacology, rooted in systems biology, is a flexible approach combining the disciplines of bioinformatics, computational analysis, and polypharmacology, which can be used to investigate the complex interactions inherent in Chinese herbal medicine (Zhang et al., 2019). Network pharmacology therefore offers the possibility of a solution to multiple element profile complexity, in which intersections and interactions of physiological proteins and other biological factors are important (Hu et al., 2019). The paradigm of network pharmacology also accelerates drug discoveries featuring as combinatorial rules and compound-gene associations (Shao Li and Zhang., 2013). In recent years, network pharmacology has been wildly employed in Chinese medicine research, including pharmacology, phytochemistry, and pharmacokinetics, providing a holistic view on the complex relationships between herbal medicine and the host (Zhao et al., 2019a; Yuan et al., 2020).

Hence, we applied this approach in an attempt to explain the mechanisms by which Danshen acts as a treatment for IBD. Compounds with oral bioavailability (OB) ≥ 30% and drug-likeness (DL) ≥ 0.18 were selected based on the Traditional Chinese Medicine System Pharmacology database and Analysis Platform (TCMSP) evaluation criteria (Jiang et al., 2019). Other supplementary compounds with potentiality, bioactivity, and experimental manipulation of which was described in “Pharmacological actions and mechanisms of Danshen for IBD treatment”, were included to construct and enrich the compound library of Danshen (Supplementary Material S1). The Traditional Chinese Medicine Integrated database (TCMID) (Huang et al., 2018) and PubChem were consulted to find and remove duplications and mismatched compounds and then all eligible compounds were validated to exist in Danshen. MOL2, SMILES, and SDF formats of each compound were captured and then uploaded to Swiss Target Prediction (Gfeller et al., 2014), STITCH (Kuhn et al., 2008), and TCMSP to retrieve predictive targets. The following databases were also searched to construct a disease library: Online Mendelian Inheritance in Man (OMIM), Therapeutic Target database (TTD), National Center for Biotechnology Information (NCBI), the Pharmacogenomics Knowledge base (PharmGKB), Malacards, and Drugbank. The entry identifiers of all targets were manually converted into Uniprot symbols for uniformity. An herb-compound-target-disease network, which was formed by intersected targets between the compound library and the disease library (Supplementary Material S2), was created using Cytoscape 3.5.1 to explore the multi-level Danshen treatment. The intrinsic protein-protein interactions (PPIs) of IBD and Danshen were visualized using STRING (Szklarczyk et al., 2019). In addition, all eligible targets were input to DAVID Bioinformatics Resources for Kyoto Encyclopedia of Genes and Genomes (KEGG) pathways and ClueGO plugin for Gene Ontology (GO) (Bindea et al., 2009).

**FIGURE 4:**
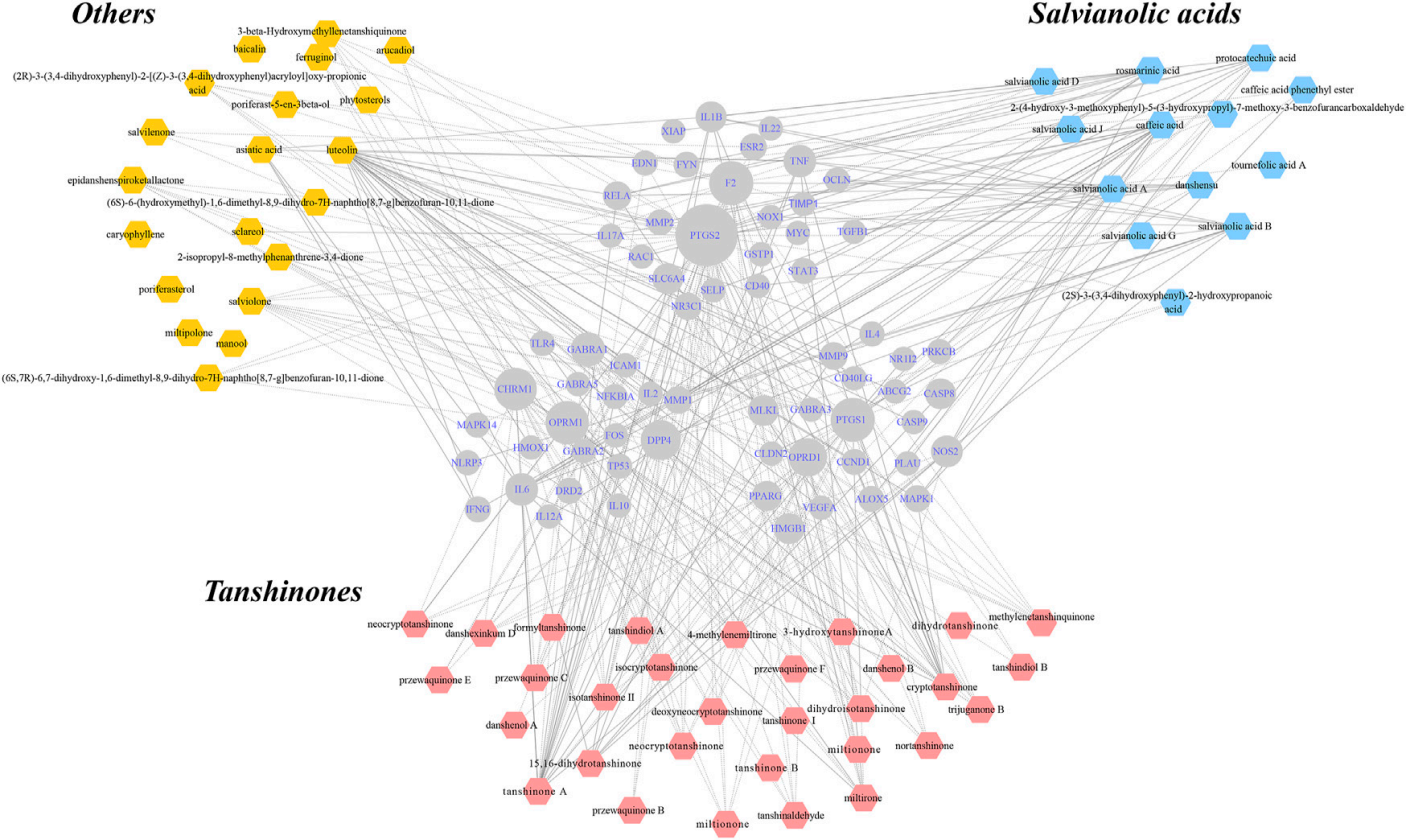
Construction of “herb-compound-target-disease” network for unmasking underlying mechanisms of Danshen for inflammatory bowel disease (IBD). Eligible compounds of Danshen were characterized within three kinds of colors while the round shapes in the center represented intersected targets both of Danshen and IBD. The scale of hubs indicated degree of targets. Solid lines indicated interactions between certain compounds and targets that were supported in the literature.

In total, 64 compounds were identified for 66 IBD-Danshen overlapping targets, reflecting a range of possible functions of Danshen in the treatment of IBD. The resulting “herb-compound-target-disease” network, which included 130 hub nodes and 124 literature-supported lines of 346 intersectional ones, represented potential targets for botanical compounds in the treatment of IBD. Each objective compound could have more than one target, which in turn could interact with various compounds (Figure 4). Overlapping targets were uploaded to yield PPIs featuring 66 hubs and 404 interactions (Supplementary Figure S1).

Meanwhile, evidence-supported functions including positive regulation of cytokine production, inflammatory response, and positive regulation of programmed cell death appeared to be affected by Danshen in Supplementary Figure S2. In addition, other processes of cell component and cell activity cooperated to exhibit pleiotropic effects, reflecting the holistic principles of Danshen treatment.

As putative targets of compounds of Danshen were included in the integrated network of multiple regulatory systems responding to extrinsic and endogenous irritants, KEGG was used to imply direct and indirect signaling transduction of Danshen’s potential action (Supplementary Material S3). The results were concentrated on TNF signaling pathway, JAK/STAT signaling pathway, TLR/NLR signaling pathway, NF-κB signaling pathway, HIF signaling pathway, PI3K/AKT/PTEN signaling pathway, and MAPK signaling pathway, all playing irreplaceable roles in the process of IBD multiply regulated by Danshen. When comparing positions of Tanshinones with Salvianolic acids in the network, some common targets, such as NF-κB, IL-6, and TNF, which were enriched in inflammatory-associated signaling pathways, were correlated with these two clusters of Danshen compounds. Meanwhile, certain specific targets, such as PPAR-γ to Salvianolic acid B, and estrogen receptors to Tanshinones, achieving in dynamic regulated functions, improved multilevel management of Danshen’s therapies of IBD.

Network Pharmacology Evaluation Method Guidance-Draft, the recent guidance for network pharmacology research, provided a systematic evaluation method (Li, 2021). Therefore, we employed this criterion to assess the network object of Danshen-IBD, especially from aspects of data retrieving, network analysis, and outcome evaluation. We performed our work to meet fundamental requirements of the Network Pharmacology Evaluation Method Guidance-Draft but need validated data, such as existing compounds, functional proteins, and further experimental verification.

## Safety of Danshen Products for IBD

Danshen and its phytochemical constituents possess multiple therapeutic efficacies and have the potential in transformation of novel drugs. During the development of pharmaceutical industry, drug safety is necessary for further research. Danshen and Danshen-containing products can be tolerated for clinical usage (Jia et al., 2019). With safety tested by a set of toxicity research on various mammals including Sprague-Dawley rats, Beagle dogs, and mice, Danshen and Danshen-containing products, offer an optional treatment of IBD with acceptable tolerance (Gao et al., 2009; Li et al., 2009; Wang et al., 2012). In 2017, a real world study, employing data of 30180 consecutive inpatients from 36 hospitals, stated that a Danshen-containing product called *Salvia Miltiorrhiza* Depside Salt for Infusion was of good tolerance by the general population (Yan et al., 2017). In the included trials in “CLINICAL TRIALS OF DANSHEN AS A TREATMENT OF UC”, side events, which best reflect safety of Danshen products on human being, had no significant difference between control groups and Danshen-intervention groups. Meanwhile, the treatment course ranged from 4 to 8 weeks, covering the general treatment course in clinic. Although Tanshione IIA exhibited potential cardiotoxicity and other severe toxicity in zebrafish embryos at high concentrations, as Danshen-containing products are mostly made by hydrophilic constituents of Danshen, there remains the declaration of safety of Danshen-containing products for IBD treatments (Wang T. et al., 2017).

## Conclusion

IBD is a chronic inflammatory disease, which induces heavy burden and lacks satisfying treatments. Although innovative drugs like monoclonal antibodies and specific agents achieve in better management and less side events, the high cost asks for economical and practical agents that are specific to IBD. Danshen is a classical herbal material frequently prescribed in Chinese medicine with a long history of over 2000 years. This herb possesses multiple therapeutic effects with low toxicity and is attracting more attention as a potential IBD treatment.

Clinical trials in which Danshen products were used supplementarily with conventional treatments indicated the efficacy of Danshen on symptoms and disease biomarkers. The Danshen products of hydrophilic cluster is used more often in clinical trials and these products like Danshen injection have shown ensured efficacy. However, as quality control of Chinese medicine injection is in challenge because of the sophistication of compounds directly entering blood, the side events can not be neglected, which asks for further evaluation on the safety of Danshen products used for IBD. Meanwhile, methodological issues including small sample sizes and flawed outcome measurements limit believability of the conclusion on Danshen’s capacity.

The compounds of Danshen are fundamentally classified by their solubility as phenolic acids and tanshinone derivatives. The development of instruments of high resolution, accuracy, and sensitivity, makes it possible to discover novel compounds of Danshen, which might have the potential to manage colitis. For instance, Savianolic acid C, one of the derivates of Savianolic acid B, has significant anti-inflammatory and anti-oxidative effects but has not been tested for colitis (Song et al., 2018). The individual compounds of Danshen with different structural features are reported to possess multiple pharmacological functions in colitis model. With deeper understand in the pathology of IBD, the anti-colitis capacity of compounds of Danshen can be explained clearly in another way. For instance, ferroptosis, which could be controlled by Tanshinone IIA, is recently reported to play an important role in IBD and will be the future field in which Danshen possesses anti-colitis capacity (Guan et al., 2020; Xu et al., 2020). Meanwhile, as a mixture of numerous compounds, Danshen exhibits its effects through multiple reactions among various compounds, different targets, and alternative biosystems. To give concise descriptions, system pharmacology approaches like network pharmacology are applied to visualize molecular mechanisms of Danshen. Although it has been developed for about 10 years, network pharmacology in Chinese medicine is still in infant to mimic the actual bio-reactions. Hence, more methods like omics are encouraged to use together to make a systematic figure of Danshen as a treatment of IBD.

With the case of Danshen in the management of IBD, the usage of medicinal herbs for complex disease shines a light on drug discovery, in which multipotent responses and uniqueness of the individuals are considered. Discovery of innovative compounds and exploring their applications are pivotal aims in the field of herbal medicine, and will contribute to the development of marketable botanical products such as Danshen injection in the future.

## Glossary

AA: Asiatic acid
BC: β-Caryophyllene
CD: Crohn’s disease
COX-2: Cyclooxygenase-2
CAT: catalase
CAT/MPO: catalase/myeloperoxidase
CRY: Crypotanshinone
CRP: C-reactive protein
CA: Caffeic acid
Danshen: Salvia miltiorrhiza Bge.
D-D: D-dimer
DT: Dihydrotanshinone
DSS: Dextran sulfate sodium
DL: D-Lactate
FIB: Plasma fibrinogen
GPx: Glutathione peroxidase
GST: Glutathione-S-transferase
GO: Gene Ontology
GSH: Glutathione
H and E: Hematoxylin and eosin stain
HIF: Hypoxia-inducible factor
IBD: Inflammatory bowel disease
iNOS: Inducible nitric oxide synthase
MUC: Mucin
MLCK: Myosin light chain kinase
MLB: Magnesium lithospermate B
MCP-1: Monocyte chemoattractant protein-1
MPV: Mean platelet volume
NOD2: Nucleotide-binding oligomerization domain-containing protein 2
NF-κB: Nuclear factor kappa B
NLR: NOD-like receptors
PLT: Serum levels of platelet
PA: Protocatechuic aldehyde
PPI: Protein-protein interaction
PAC: Protocatechuic acid
RCT: Random control trial
RA: Rosmarinic acid
RAME: Rosmarinic acid methyl ester
ROS: Reactive oxygen species
SOD: Superoxide dismutase
SAA: Salvianic acid A: Salvianolic acid A
SAB: Salvianolic acid B
TIIA: Tanshinone IIA
TIIB: Tanshinone IIB
TNBS: 2,4,6-Trinitrobenzene sulfonic acid
TCM: Traditional Chinese medicine
TLR: Toll-like receptors
TJ: Tight junction
TCMSP: Traditional Chinese Medicine System Pharmacology database and Analysis Platform
TGF-β: Transforming growth factor β
UC: Ulcerative colitis
ZO: Zonula occludens.
