# Supplementary material for: Salvia miltiorrhiza Bge. (Danshen) for Inflammatory Bowel Disease: Clinical Evidence and Network Pharmacology-Based Strategy for Developing Supplementary Medical Application
Source: Front Pharmacol. 2022 Jan 19;12:741871. doi: 10.3389/fphar.2021.741871 (PMC8807566; doi:10.3389/fphar.2021.741871)
Supplement: Supplementary file 1 [file DataSheet1.ZIP › DataSheet1 .docx]

**Supporting Information**

**Salvia miltiorrhiza** **Bge. (Danshen) for inflammatory bowel disease: clinical evidence and network pharmacology-based strategy for developing supplementary medical application**

Siyuan Zhang^1^, Hua Luo^1^, Shiyi Sun^2^, Yating Zhang^2^, Jiaqi Ma^2^, Yuting Lin^2^, Lin Yang^1^, Dechao Tan^1^, Chaomei Fu^2^, Zhangfeng Zhong^1^*, Yitao Wang^1^*

^1^ Macau Center for R&D in Chinese Medicine, Institute of Chinese Medical Sciences, University of Macau, Taipa, Macao, China

^2^ Pharmacy School, Chengdu University of Traditional Chinese Medicine, Liutai Road, Chengdu, China

^*^Correspondence: Dr. Zhangfeng Zhong: zhangfengzhong@um.edu.mo; Prof. Yitao Wang: [ytwang@um.edu.mo](mailto:ytwang@um.edu.mo)

Siyuan Zhang: [yb97506@um.edu.mo](mailto:yb97506@um.edu.mo)

Hua Luo: [yb87518@um.edu.mo](mailto:yb87518@um.edu.mo)

Shiyi Sun: ssy750309@163.com

Jiaqi Ma: [mjq1493424378@163.com](mailto:mjq1493424378@163.com)

Yating Zhang: rhizoma123@sina.com

Yuting Lin: lyt3176117016@163.com

Lin Yang: [mb95803@um.edu.mo](mailto:mb95803@um.edu.mo)

Dechao Tan: [dechaotan@foxmail.com](mailto:dechaotan@foxmail.com)

Chaomei Fu: [chaomeifu@126.com](mailto:chaomeifu@126.com)

**FIGURE S1| Protein-Protein interactions of overlapping targets accounting for potential interactions.** The hubs indicated different protein targets and the lines indicated their interactions.

**FIGURE S2| GO results of overlapping targets.** The round shape indicated alternative GO functions of potentiality while similar colors showed a close relationship among clustered functions.

**TABLE S1|** **Eligible compounds of Danshen.**

**TABLE S2|** **Targets of Danshen compounds and IBD.**

**TABLE S3|** **KEGG pathway enrichment of Danshen-IBD targets.**
