# Supplementary figures and images for: Salvia miltiorrhiza Bge. (Danshen) for Inflammatory Bowel Disease: Clinical Evidence and Network Pharmacology-Based Strategy for Developing Supplementary Medical Application
Source: Front Pharmacol. 2022 Jan 19;12:741871. doi: 10.3389/fphar.2021.741871 (PMC8807566; doi:10.3389/fphar.2021.741871)

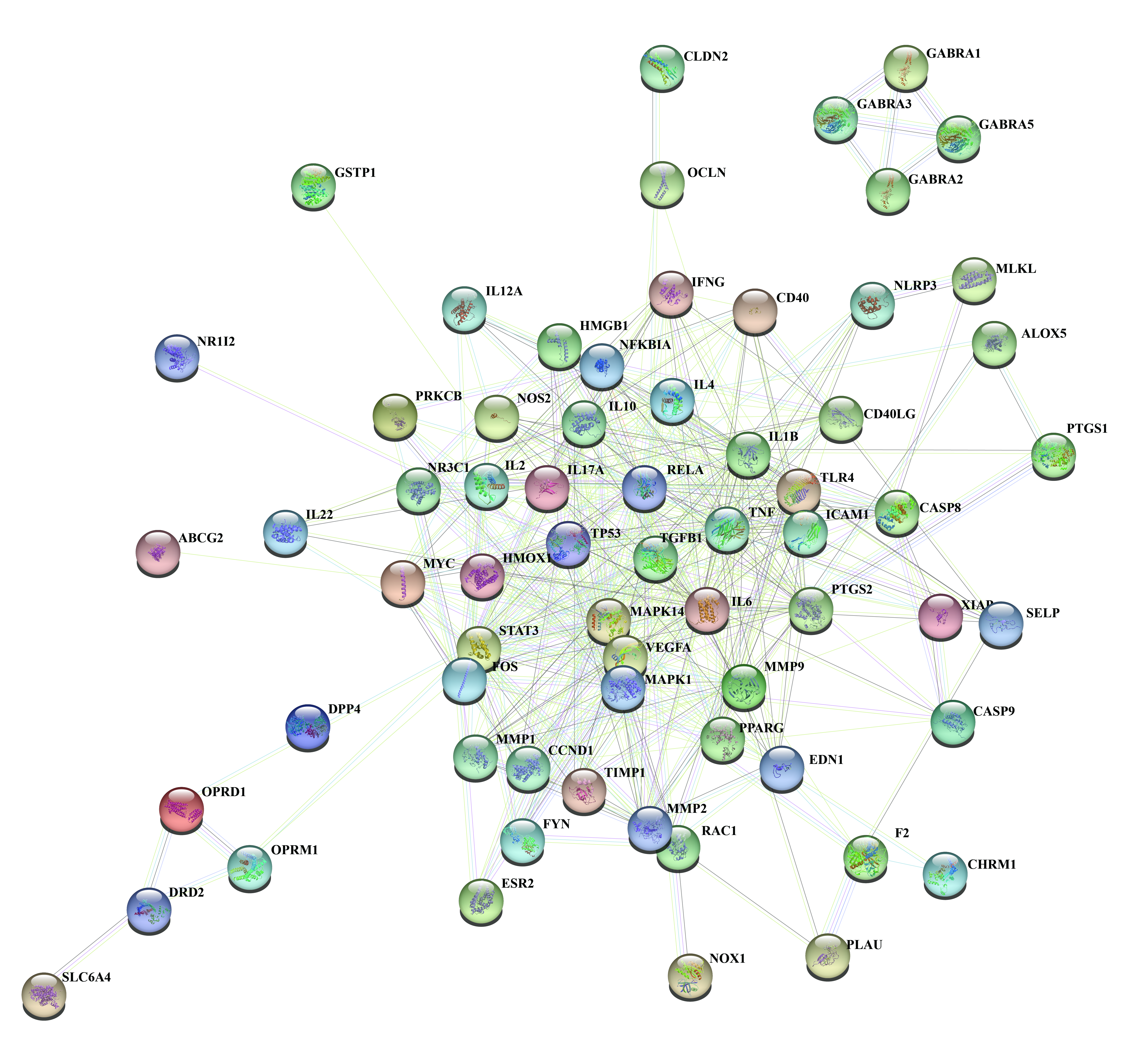

Supplement: Supplementary file 1 [file DataSheet1.ZIP › FIGURE S1.jpg]

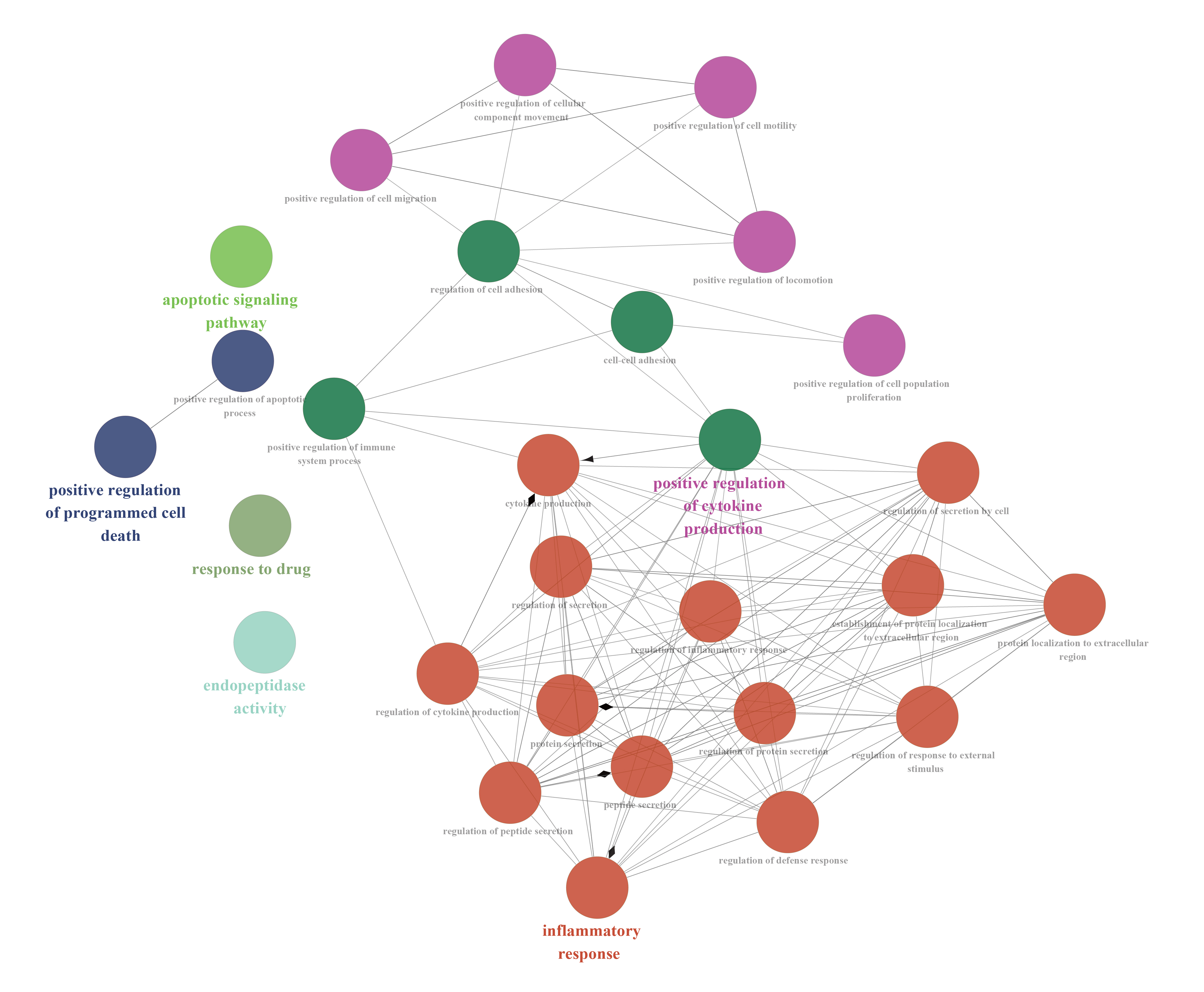

Supplement: Supplementary file 1 [file DataSheet1.ZIP › FIGURE S2.jpg]
